# Supplementary material for: Metabolome-driven microbiome assembly in ginger (Zingiber officinale) enhances nutrient cycling and crop yield through keystone taxa
Source: Commun Biol. 2025 Nov 10;8:1547. doi: 10.1038/s42003-025-08910-2 (PMC12603305; doi:10.1038/s42003-025-08910-2)
Supplement: Supplementary file 1 — Supplementary Information [file 42003_2025_8910_MOESM1_ESM.pdf]

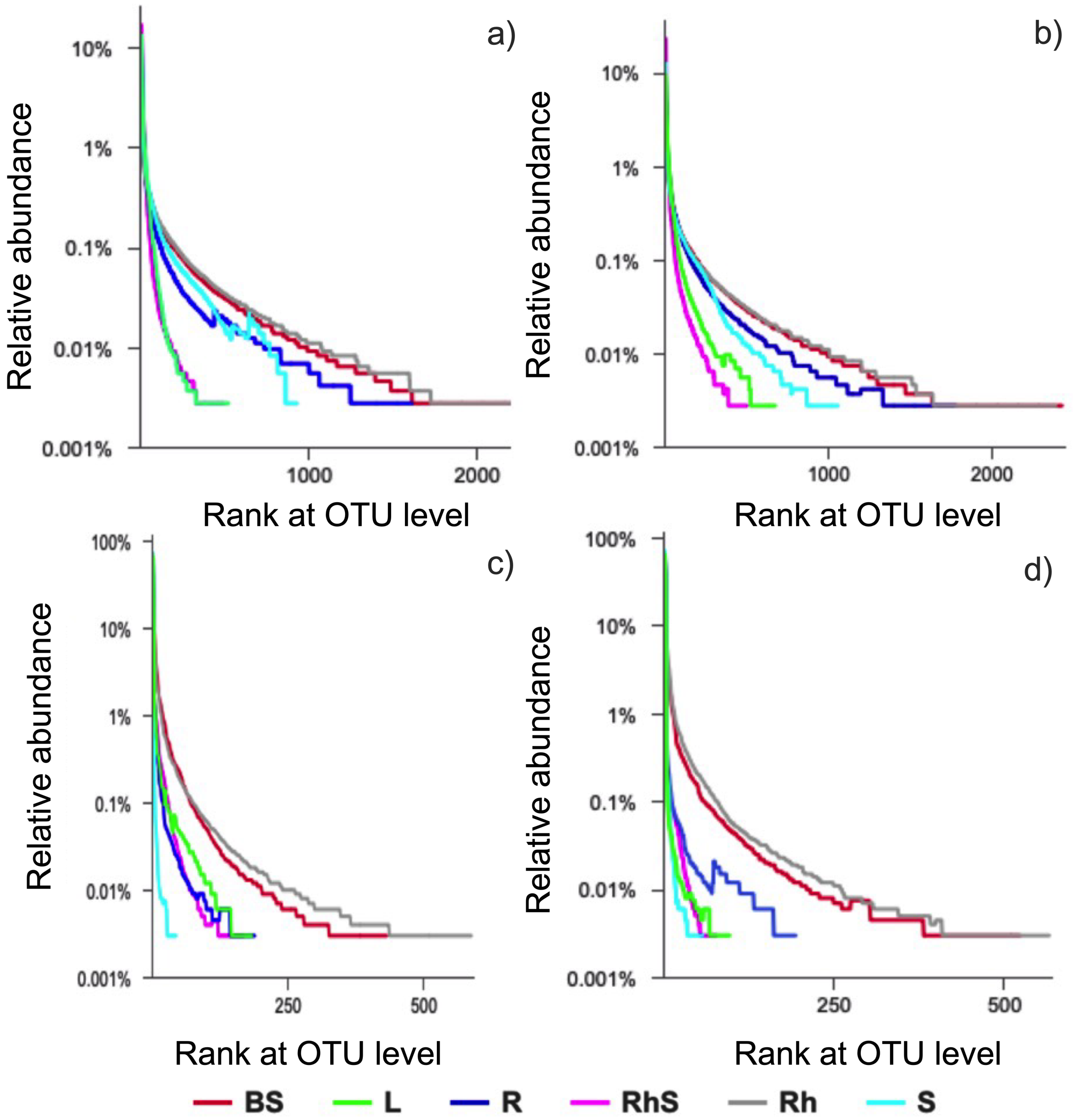

Supplementary Figure 1 | Rank abundance curves of bacterial and fungal communities across ginger niches.

Rank-abundance plots show OTU distribution for bacterial/archaeal (a, b) and fungal (c, d) communities in two ginger varieties. The x-axis denotes OTU rank; the y-axis indicates relative abundance. Curve length reflects OTU richness per sample.

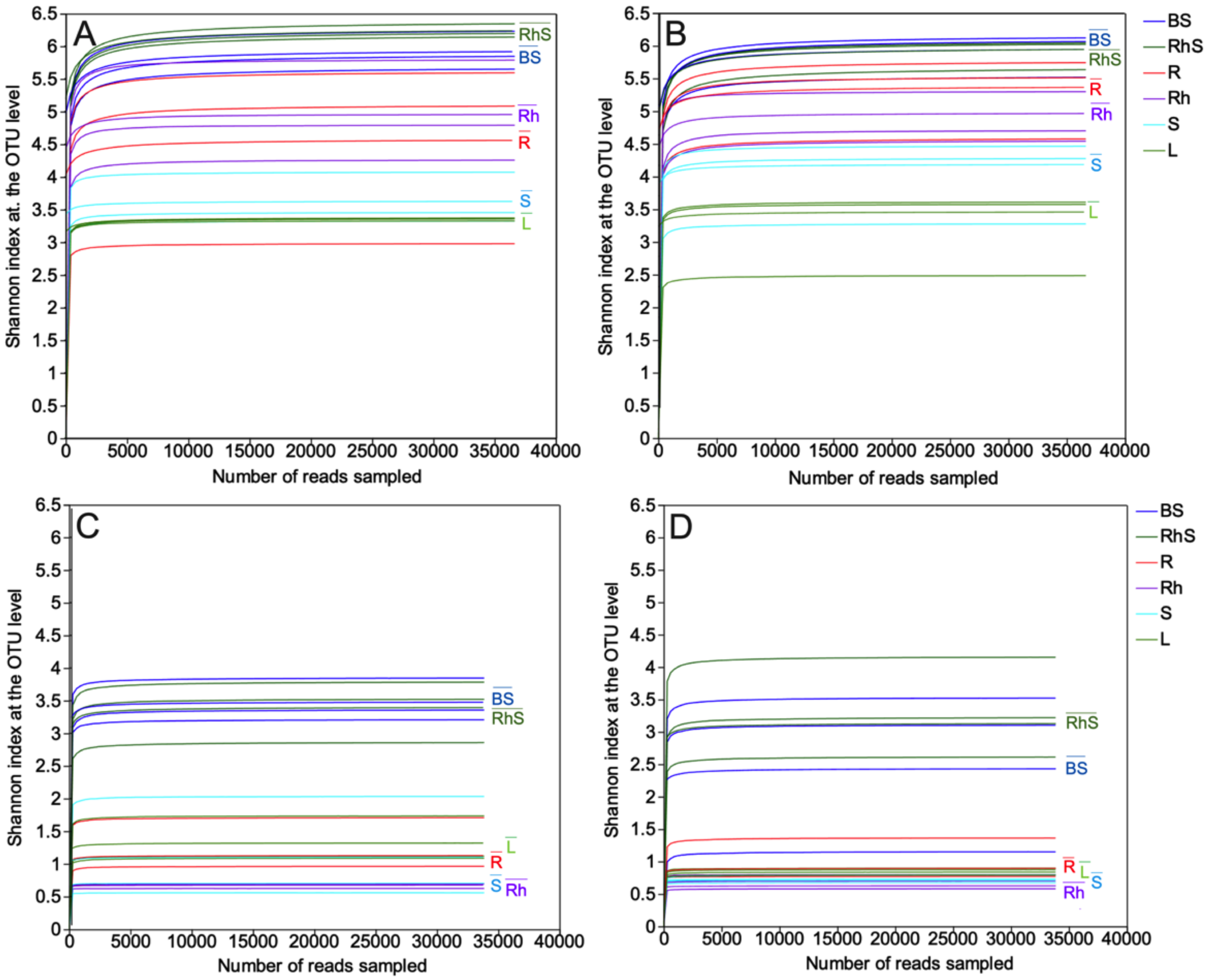

Supplementary Figure 2 | Shannon rarefaction curves of bacterial and fungal communities in ginger niches.

Shannon diversity rarefaction curves for bacterial/archaeal (a, b) and fungal (c, d) communities across microbial niches in two ginger varieties. Based on repeated random subsampling, the curves show OTU-level diversity (97% similarity) versus sequencing depth.
