# Supplementary material for: Metabolome-driven microbiome assembly in ginger (Zingiber officinale) enhances nutrient cycling and crop yield through keystone taxa
Source: Commun Biol. 2025 Nov 10;8:1547. doi: 10.1038/s42003-025-08910-2 (PMC12603305; doi:10.1038/s42003-025-08910-2)
Supplement: Supplementary file 2 — Description of Additional Supplementary Files [file 42003_2025_8910_MOESM2_ESM.pdf]

Description of Additional Supplementary Files

File name: Supplementary Data 1

Description: Supplementary Data of Fig.1

File name: Supplementary Data 2

Description: Supplementary Data of Fig.2

File name: Supplementary Data 3

Description: Supplementary Data of Fig. 3

File name: Supplementary Data 4

Description: Supplementary Data of Fig. 4

File name: Supplementary Data 5

Description: Supplementary Data of Fig. 5

File name: Supplementary Data 6

Description: Supplementary Data of Fig. 6

File name: Supplementary Data 7

Description: Supplementary Data of Fig. 7

File name: Supplementary Table 1

Description: Physicochemical properties of microbial niches in two ginger varieties. Includes soil and tissue nutrient data across BS (bulk soil), RhS (rhizosphere soil), R (roots), Rh (rhizomes), S (stems), and L (leaves) for both ginger varieties.
