# Supplementary material for: Metabolome-driven microbiome assembly in ginger (Zingiber officinale) enhances nutrient cycling and crop yield through keystone taxa
Source: Commun Biol. 2025 Nov 10;8:1547. doi: 10.1038/s42003-025-08910-2 (PMC12603305; doi:10.1038/s42003-025-08910-2)
Supplement: Supplementary file 11 — Reporting-summary [file 42003_2025_8910_MOESM11_ESM.pdf]

nature portfolio

Corresponding author(s): Ramon Santos-Bermudez; Nayanci Portal-Gonzalez

Last updated by author(s): 2025/09/07

Reporting Summary

Nature Portfolio wishes to improve the reproducibility of the work that we publish. This form provides structure for consistency and transparency in reporting. For further information on Nature Portfolio policies, see our Editorial Policies and the Editorial Policy Checklist.

Statistics

For all statistical analyses, confirm that the following items are present in the figure legend, table legend, main text, or Methods section.

n/a	Confirmed	
☐	☒	The exact sample size (*n*) for each experimental group/condition, given as a discrete number and unit of measurement
☐	☒	A statement on whether measurements were taken from distinct samples or whether the same sample was measured repeatedly
☐	☒	The statistical test(s) used AND whether they are one- or two-sided<br>*Only common tests should be described solely by name; describe more complex techniques in the Methods section.*
☐	☒	A description of all covariates tested
☐	☒	A description of any assumptions or corrections, such as tests of normality and adjustment for multiple comparisons
☐	☒	A full description of the statistical parameters including central tendency (e.g. means) or other basic estimates (e.g. regression coefficient) AND variation (e.g. standard deviation) or associated estimates of uncertainty (e.g. confidence intervals)
☐	☒	For null hypothesis testing, the test statistic (e.g. *F*, *t*, *r*) with confidence intervals, effect sizes, degrees of freedom and *P* value noted<br>*Give P values as exact values whenever suitable.*
☐	☒	For Bayesian analysis, information on the choice of priors and Markov chain Monte Carlo settings
☒	☐	For hierarchical and complex designs, identification of the appropriate level for tests and full reporting of outcomes
☐	☒	Estimates of effect sizes (e.g. Cohen's *d*, Pearson's *r*), indicating how they were calculated

Our web collection on statistics for biologists contains articles on many of the points above.

Software and code

Policy information about availability of computer code

Data collection	No software was used
Data analysis	Most analyses were conducted using the online Majorbio Cloud Platform (https://cloud.majorbio.com), which integrates open-source bioinformatics tools. Microbial diversity analysis and co-occurrence network construction were performed using embedded implementations of QIIME2 (version 2023.2), LEfSe (Galaxy/Huttenhower implementation), and Cytoscape (version 3.10.0). Functional predictions were carried out using the FAPROTAX and FUNGuild databases. Metabolomic data were processed with Compound Discoverer 3.3 (Thermo Fisher Scientific) and visualized using MetaboAnalyst 5.0. Experimental validation of metabolite effects and final figure assembly were both performed using GraphPad Prism (version 10.5.0). No custom code was used.

For manuscripts utilizing custom algorithms or software that are central to the research but not yet described in published literature, software must be made available to editors and reviewers. We strongly encourage code deposition in a community repository (e.g. GitHub). See the Nature Portfolio guidelines for submitting code & software for further information.

Data

Policy information about availability of data

All manuscripts must include a data availability statement. This statement should provide the following information, where applicable:

- Accession codes, unique identifiers, or web links for publicly available datasets
- A description of any restrictions on data availability
- For clinical datasets or third party data, please ensure that the statement adheres to our policy

The datasets generated and analyzed in this study are publicly available and comply with community standards for data deposition. Raw sequence data (16S rRNA for bacteria and ITS for fungi) generated via Illumina sequencing have been deposited in the NCBI Sequence Read Archive (SRA) under BioProject accessions PRJNA1035274 (bacteria) and PRJNA1035275 (fungi). Metabolomic datasets are available through the BIG Submission platform (BIG SUB) at the National Genomics Data Center (NGDC) under Bioproject PRJCA023529 (OMIX ID: OMIX005846; accessible at [https://ngdc.cncb.ac.cn/omix/release/OMIX005846]. Processed OTU tables, statistical analyses, and metabolite profiles are provided as Supplementary Data Files accompanying this manuscript. All other data supporting the findings of this study are included in the article and its supplementary materials.

Research involving human participants, their data, or biological material

Policy information about studies with human participants or human data. See also policy information about sex, gender (identity/presentation), and sexual orientation and race, ethnicity and racism.

Reporting on sex and gender	*Use the terms sex (biological attribute) and gender (shaped by social and cultural circumstances) carefully in order to avoid confusing both terms. Indicate if findings apply to only one sex or gender; describe whether sex and gender were considered in study design; whether sex and/or gender was determined based on self-reporting or assigned and methods used.*<br>*Provide in the source data disaggregated sex and gender data, where this information has been collected, and if consent has been obtained for sharing of individual-level data; provide overall numbers in this Reporting Summary. Please state if this information has not been collected.*<br>*Report sex- and gender-based analyses where performed, justify reasons for lack of sex- and gender-based analysis.*
Reporting on race, ethnicity, or other socially relevant groupings	*Please specify the socially constructed or socially relevant categorization variable(s) used in your manuscript and explain why they were used. Please note that such variables should not be used as proxies for other socially constructed/relevant variables (for example, race or ethnicity should not be used as a proxy for socioeconomic status).*<br>*Provide clear definitions of the relevant terms used, how they were provided (by the participants/respondents, the researchers, or third parties), and the method(s) used to classify people into the different categories (e.g. self-report, census or administrative data, social media data, etc.)*<br>*Please provide details about how you controlled for confounding variables in your analyses.*
Population characteristics	*Describe the covariate-relevant population characteristics of the human research participants (e.g. age, genotypic information, past and current diagnosis and treatment categories). If you filled out the behavioural & social sciences study design questions and have nothing to add here, write "See above."*
Recruitment	*Describe how participants were recruited. Outline any potential self-selection bias or other biases that may be present and how these are likely to impact results.*
Ethics oversight	*Identify the organization(s) that approved the study protocol.*

Note that full information on the approval of the study protocol must also be provided in the manuscript.

Field-specific reporting

Please select the one below that is the best fit for your research. If you are not sure, read the appropriate sections before making your selection.

☒ Life sciences ☐ Behavioural & social sciences ☐ Ecological, evolutionary & environmental sciences

For a reference copy of the document with all sections, see nature.com/documents/nr-reporting-summary-flat.pdf

Life sciences study design

All studies must disclose on these points even when the disclosure is negative.

Sample size	No formal statistical method was used to predetermine sample size. Instead, sample sizes were selected based on common practices in plant–microbiome studies and the logistical feasibility of obtaining biological replicates across all treatment groups. For each tissue type and ginger variety, three biological replicates were analyzed, which is generally accepted to ensure reproducibility and statistical power in multi-omics comparisons. These sample sizes were sufficient to detect significant differences in microbial community structure, metabolite profiles, and their correlations.
Data exclusions	No data were excluded from the analyses. All samples and data points met the quality control thresholds defined prior to analysis, including sequencing depth, metabolite detection limits, and replicate consistency. No pre-established exclusion criteria were triggered during data processing.

Replication	All experiments were conducted with at least three biological replicates per condition. Key findings were consistently observed across replicates, including microbial community shifts, metabolite profiles, and microbial-metabolite associations. No findings were excluded due to irreproducibility.
Randomization	Randomization did not apply to this study. Plants were grown under field conditions, and samples were collected from predefined plant compartments (bulk soil, rhizosphere, rhizome, root, stem, and leaf) across two ginger varieties. All efforts were made to minimize environmental and handling bias by sampling equivalent tissues at the same developmental stage and location.
Blinding	Blinding was not applicable in this study. Sample identity (variety and tissue type) was inherent to the experimental design and could not be concealed during collection or analysis. However, data analysis was conducted using automated and objective computational pipelines to minimize potential investigator bias.

Reporting for specific materials, systems and methods

We require information from authors about some types of materials, experimental systems and methods used in many studies. Here, indicate whether each material, system or method listed is relevant to your study. If you are not sure if a list item applies to your research, read the appropriate section before selecting a response.

Materials & experimental systems

n/a	Involved in the study
☒	☐ Antibodies
☒	☐ Eukaryotic cell lines
☒	☐ Palaeontology and archaeology
☒	☐ Animals and other organisms
☒	☐ Clinical data
☒	☐ Dual use research of concern
☐	☒ Plants

Methods

n/a	Involved in the study
☒	☐ ChIP-seq
☒	☐ Flow cytometry
☒	☐ MRI-based neuroimaging

Dual use research of concern

Policy information about dual use research of concern

Hazards

Could the accidental, deliberate or reckless misuse of agents or technologies generated in the work, or the application of information presented in the manuscript, pose a threat to:

No	Yes
☒	☐ Public health
☒	☐ National security
☒	☐ Crops and/or livestock
☒	☐ Ecosystems
☒	☐ Any other significant area

Experiments of concern

Does the work involve any of these experiments of concern:

No	Yes
☒	☐ Demonstrate how to render a vaccine ineffective
☒	☐ Confer resistance to therapeutically useful antibiotics or antiviral agents
☒	☐ Enhance the virulence of a pathogen or render a nonpathogen virulent
☒	☐ Increase transmissibility of a pathogen
☒	☐ Alter the host range of a pathogen
☒	☐ Enable evasion of diagnostic/detection modalities
☒	☐ Enable the weaponization of a biological agent or toxin
☒	☐ Any other potentially harmful combination of experiments and agents

Plants

Seed stocks	Ginger (Zingiber officinale) specimens were obtained from two agricultural sites in Shandong Province, China, agricultural sites. Field-grown plants representing distinct local varieties were sampled during the 2023 growing season. Each sample was collected under sterile conditions and immediately transported on ice to the laboratory for processing. No commercial seed stock center or catalogue number was applicable.
Novel plant genotypes	Variety identity was determined based on farmer records and confirmed by morphological traits and metabolite profiles.
Authentication	Plant materials were authenticated by comparative analysis of morphological characteristics and metabolite profiling across multiple replicates. Although formal genotyping was not conducted, the consistency in biochemical profiles and accompanying metadata affirmed each plant variety's identity and integrity. Transgenic lines were not employed in this study, and all samples were processed per standardized collection protocols to ensure traceability.
